# Over-the-Air Testing of a Massive MIMO Antenna with a Full-Rank Channel Matrix

**DOI:** 10.3390/s22031240

**Published:** 2022-02-06

**Authors:** Kazuhiro Honda

**Affiliations:** Graduate School of Engineering, Toyama University, 3190 Gofuku, Toyama 930-8555, Japan; hondak@eng.u-toyama.ac.jp; Tel.: +81-76-445-6759

**Keywords:** massive multiple-input multiple-output (MIMO) antenna, over-the-air (OTA) testing, channel matrix, full-rank, fading emulator

## Abstract

This paper presents an over-the-air testing method in which a full-rank channel matrix is created for a massive multiple-input multiple-output (MIMO) antenna system utilizing a fading emulator with a small number of scatterers. In the proposed method, in order to mimic a fading emulator with a large number of scatterers, the scatterers are virtually positioned by rotating the massive MIMO antenna. The performance of a 64-element quasi-half-wavelength dipole circular array antenna was evaluated using a two-dimensional fading emulator. The experimental results reveal that a large number of available eigenvalues are obtained from the channel response matrix, confirming that the proposed method, which utilizes a full-rank channel matrix, can be used to assess a massive MIMO antenna system.

## 1. Introduction

Global commercial services for ultra-high speed fifth-generation (5G) mobile communication using multiple-input multiple-output (MIMO) systems are currently available [1,2]. One of the possible solutions to significantly enhance the channel capacity of MIMO systems is to utilize a large number of antenna elements for both the base station (BS) and the mobile station (MS). Such a system is called a massive MIMO system [3].

Most of the activity so far undertaken in developing massive MIMO systems has been directed toward providing large-scale MIMO antennas at the BS, with antennas comprising more than 100 antenna elements [4,5], and there are few reports of doing something similar at the MS [6]. The author is currently developing a method to achieve a large-scale MIMO antenna system that maintains an invariable channel capacity over the full-azimuth at the MS, such as, for example, a connected ground-based or flying car [7].

The usual technique for evaluating the performance of MIMO antennas with multipath fading channels is to do Monte Carlo simulation where several scatterers are placed on a circle [8,9]. This is known as the Clarke model or ring model. Using this model, the number of scatterers required to simulate the full-rank property of the channel matrix for a massive MIMO system is greater than the number of subchannels.

To analyze the capability of the developed antenna [7], the author proposed a Monte Carlo simulation with randomly arranged scatterers [10]. A small number of differently positioned scatterers were set for each BS antenna using random numbers, confirming that the channel matrix created can achieve full-rank status similar to conventional Monte Carlo simulation. However, a large number of scatterers are necessary to emulate a lot of channels.

A legitimate manner of assessing the performance of a fabricated massive MIMO antenna is to test it in the field [11]. However, with field testing, the measurements are neither repeatable nor controllable, and, moreover, the measurement process is considerably time-consuming and labor-intensive. Hence, over-the-air (OTA) testing, which evaluates the ability of a MIMO antenna by reproducing a realistic multipath radio propagation environment in the laboratory, is essential.

A proper OTA testing method for massive MIMO antennas is required to accelerate the development and optimization of the antenna [12]. As many massive MIMO BS antennas have been developed, radiating evaluation methods for massive MIMO BS antennas using fading emulators have also been investigated [13,14]. Many BS antennas comprise a two-dimensional planar array antenna with a number of patch antennas. Consequently, a large number of scatterers are placed in a limited direction with respect to the OTA apparatus and scatterers are selected from among them for the assessment.

On the other hand, an OTA testing method for massive MIMO MS antennas is not currently available. A fading emulator with a small number of scatterers placed on a circle has been adopted for proper OTA testing of a handset MS comprising a few MIMO antenna elements [15,16]. In the standard method utilizing a fading emulator, the arrangement of the scatterers depends on the number of subchannels, indicating that a large number of scatterers are necessary to assess the performance of a massive MIMO system with a full-rank channel matrix. Therefore, because of the size and cost of the equipment, the standard method is not effective for massive MIMO MS antennas.

The author proposed an OTA evaluation method for a massive MIMO antenna that creates a full-rank channel matrix [17]. The results of the Monte Carlo simulation, which included simulation of the proposed OTA testing method, revealed that even though the channel model comprised a limited number of scatterers, a full-rank channel matrix can be created. However, an experimental verification of this has not been done.

This paper presents an experimental verification of the proposed method utilizing a two-dimensional fading emulator with a small number of scatterers. In the OTA measurements, the massive MIMO MS antenna is located at the center of the fading emulator and is rotated depending on the measured channel response at the BS antenna, in such a way that a small number of scatterers are equivalent to a much larger number of scatterers. The experimental results showed that the number of available eigenvalues is greater than that obtained with the previous method.

## 2. Measurement Method of the Full-Rank Channel

In a previous Monte Carlo simulation with a uniform arrangement of scatterers, which represented the secondary wave source, *N* × *M* channel responses were calculated to form Equation (1).

(1) $$H_{S}=\left[\begin{array}{cccccc} h_{11} & h_{12} & \cdots & h_{1K_{m}} & \cdots & h_{1M}\\ h_{21} & h_{22} & \cdots & h_{2K_{m}} & \cdots & h_{2M}\\ \vdots & \vdots & \ddots & \vdots & \ddots & \vdots \\ h_{N1} & h_{N2} & \cdots & h_{NK_{m}} & \cdots & h_{NM} \end{array}\right]$$

where *K_m_* indicates the number of actual scatterers. *M* and *N* denote the number of elements in the BS and MS, respectively. Furthermore, the author assumed that the number of elements at the BS is equal to that at the MS, that is, *M* = *N*.

In Equation (1), because all the signals from the *M* elements at BS overlap with each other at the same location, the number of columns that satisfy linear independence is equal to *K_m_* in the channel model. Consequently, assuming that *K_m_* is less than *M*, Equation (1) is transformed into Equation (2) using diagonalization.

(2) $$H_{S}=\left[\begin{array}{ccccccc} h_{11} & h_{12} & \cdots & h_{1K_{m}} & 0 & \cdots & 0\\ h_{21} & h_{22} & \cdots & h_{2K_{m}} & 0 & \cdots & 0\\ \vdots & \vdots & \ddots & \vdots & \vdots & \ddots & \vdots \\ h_{N1} & h_{N2} & \cdots & h_{NK_{m}} & 0 & \cdots & 0 \end{array}\right]$$

Therefore, the eigenvalue vector obtained using singular value decomposition (SVD) is denoted by Equation (3).

(3) $$\Lambda =\left[\begin{array}{ccccccc} \lambda_{1} & \lambda_{2} & \cdots & \lambda_{K_{m}} & 0 & \cdots & 0 \end{array}\right]$$

The rank of Equation (2) equals *K_m_* which is less than *M*, indicating a rank-deficient status. Consequently, the number of eigenvalues, that is, the number of channels, observed is only *K_m_*. Hence, a large number of scatterers, greater than *M*, are necessary to obtain full-rank status with rank (**H***_S_*) = *M*.

The method of randomly arranged scatterers, in which a limited number of scatterers are arranged to simulate each BS element, was proposed [10]. The required number of scatterers to generate the Rayleigh fading environment for one BS element is small. However, the total number of scatterers is the product of *M* and *K_m_*. Consequently, for OTA testing of a massive MIMO system incorporating the method of randomly arranged scatterers, a small number of scatterers must be selected from among the large number of scatterers on the circle of the fading emulator. Otherwise, the actual scatterers need to be relocated for each BS. Hence, the OTA testing implemented using the method of randomly arranged scatterers is extremely labor-intensive process compared with the standard OTA testing method.

**Figure 1:**
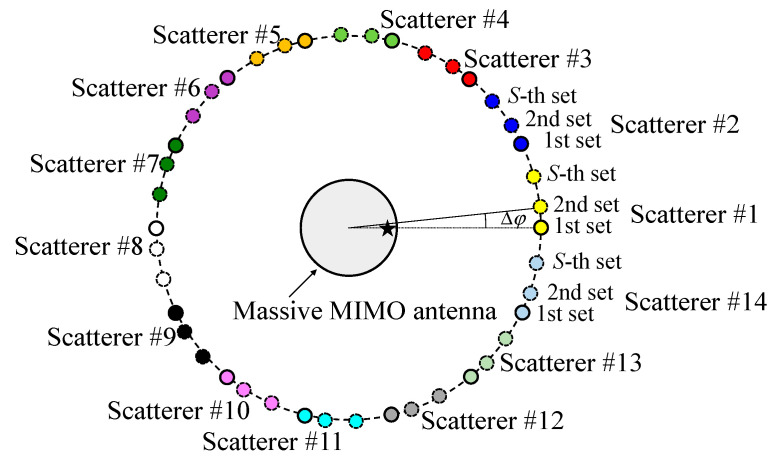
Configuration of the scatterers for evaluating the massive MIMO system.

The author proposed an OTA testing method in which the scatterers are virtually formed emulating a large number of scatterers [17]. Figure 1 shows the configuration of the proposed fading emulator to enable a full-rank channel matrix for a massive MIMO antenna. In Figure 1, *K_m_*, that is, the number of scatterers in the 1st set, is 14 which is sufficient to produce a Rayleigh fading environment. The 2nd to *S*-th sets of scatterers are virtually placed, where *S* indicates the number of scatterer sets.

The angular intervals between the *i*-th and (*i* + 1)-th sets of scatterers, Δ*φ*, as shown in Figure 1, are the same. Therefore, each set of scatterers is formed by rotating the 1st set of scatterers. Consequently, the placement of each of scatterer differs, and a large number of scatterers can be emulated, with the expectation that measurement with a full-rank property of the channel matrix can be achieved.

The most important parameter in the measurement is the number of scatterer sets which depends on the number of actual scatterers. Independent paths via each BS antenna are generated by orthogonal initial phase sets at the actual scatterers. Hence, the maximum number of BS elements emulated in accordance with each set of scatterers is the same as the number of actual scatterers. In order to achieve a full-rank matrix, *S* must be adjusted to be greater than *M* divided by *K_m_*. Another important parameter is Δ*φ*. If Δ*φ* is small, the possibility of generating different paths from adjacent sets of scatterers is small. In this paper, Δ*φ* is set to equal angular intervals, and it is calculated as follows:

(4) $$\Delta \varphi =\frac{360}{K_{m}}\frac{1}{S}$$

**Figure 2:**
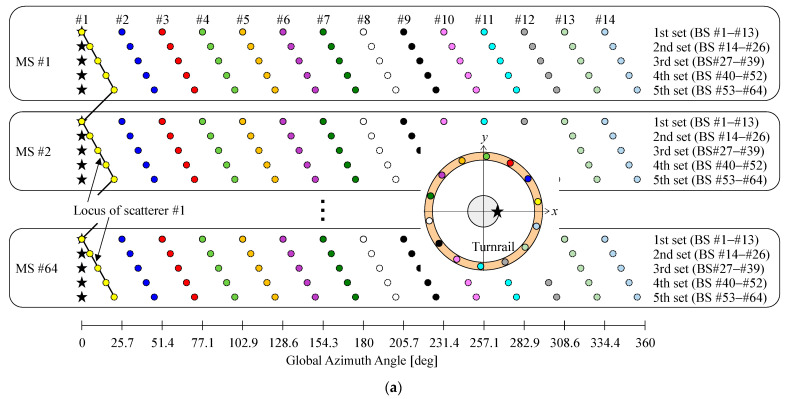

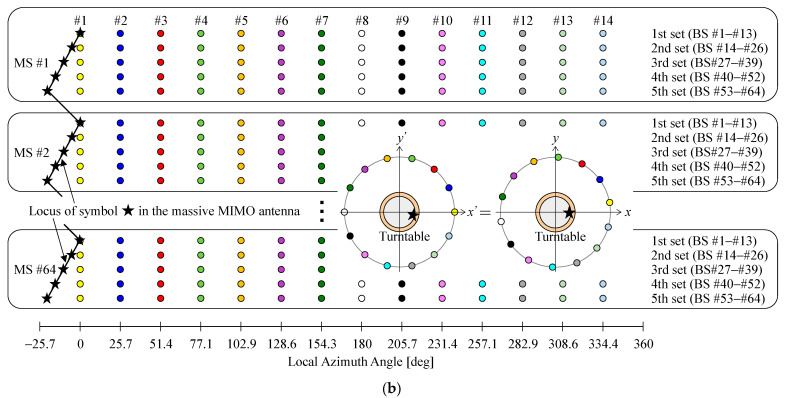
Massive MIMO OTA apparatus: (**a**) with the turn rail; (**b**) with the turntable.

There are two ways to construct the fading emulator embodied using the proposed method. One of the possible ways is to rotate the turn rail on which the actual scatterers are located, as shown in the insert in Figure 2a, which is the same structure shown in Figure 1. Another is that the massive MIMO antenna, which is placed at the center of the turntable, is rotated, as illustrated on the left hand side of the inset in Figure 2b. In this case, the rotation target, that is, the massive MIMO antenna, is different to that of Figure 1, but Figure 1 and Figure 2b have the same benefit of achieving a full-rank channel matrix measurement, as explained in below.

It is known that with multiple probe antenna based methods, such as those with fading emulators, the repeatability and controllability of the radio propagation environment are superior to those obtained with other OTA testing methods, such as reverberation chamber based methods or two-stage methods [18]. In OTA assessment using a fading emulator, the MIMO channel response between the *m*-th BS antenna element and *n*-th MS antenna element is measured individually, taking advantage of the high time correlation characteristics of the apparatus.

Figure 2 shows the relationship between the channel response measured, the azimuth angle of the actual scatterers, and the azimuth angle of the massive MIMO antenna in the case of a 64 × 64 MIMO system, with *K_m_* = 14, and *S* = 5. The symbols in Figure 2 are associated with Figure 1. The circles indicate the positions of the scatterers, whereas the star symbol denotes the azimuth angle of the massive MIMO antenna, which starts from 0° in the measurements.

In Figure 2a, the massive MIMO MS antenna is fixed at the center of the fading emulator, and the actual scatterers are moved by rotating the turn rail. Accordingly, the global azimuth angle of the scatterers is varied depending on the measured channel response for the *m*-th BS antenna. The black line indicates the locus of scatterer #1.

The channel responses from BS #1 to BS #13 are measured with the 1st set of scatterers in place. Then, the turn rail is rotated by Δ*φ*, and the channel responses from BS #14 to BS #26 are measured with the 2nd set of scatterers in place. By repeating this procedure, the channel responses of the *n*-th MS antenna are fulfilled. Moreover, this method applies to all MS antennas, resulting in a full-rank channel response matrix. 

In contrast, in Figure 2b, the actual scatterers remain stationary, and the turntable with the massive MIMO MS antenna is rotated. Consequently, the local azimuth angle of the massive MIMO MS antenna is changed corresponding to the measured channel response for the *m*-th BS antenna. The black line shows the locus of the star symbol expressing the angle of the massive MIMO antenna. However, the global azimuth angle of the MIMO antenna is fixed during OTA testing. When the local azimuth angle is transformed so that the global azimuth angle is 0°, as shown on the right hand side of the inset in Figure 2b, it becomes the same as in Figure 2a. Eventually, the actual scatterers are virtually positioned in different locations.

The channel responses from BS #1 to BS #13 are measured. Then, the turntable is rotated by −Δ*φ*, and the channel responses from BS #14 to BS #26 are obtained. By repeating this operation, the channel responses of the *n*-th MS antenna are satisfied. Furthermore, this is done for all MS antennas, demonstrating that the channel matrix is full-rank status.

## 3. Results and Discussion

### 3.1. Analytical Results

To verify the proposed OTA method, Monte Carlo simulation of a massive MIMO antenna was conducted. The massive MIMO antenna comprises a 64-element quasi-half-wavelength dipole MIMO circular array antenna at 5 GHz to exclude the effect caused by electromagnetic mutual coupling. The array antenna was arranged with equal angular intervals. The radiation pattern of the massive MIMO antenna was calculated by the method of moments.

**Figure 3:**
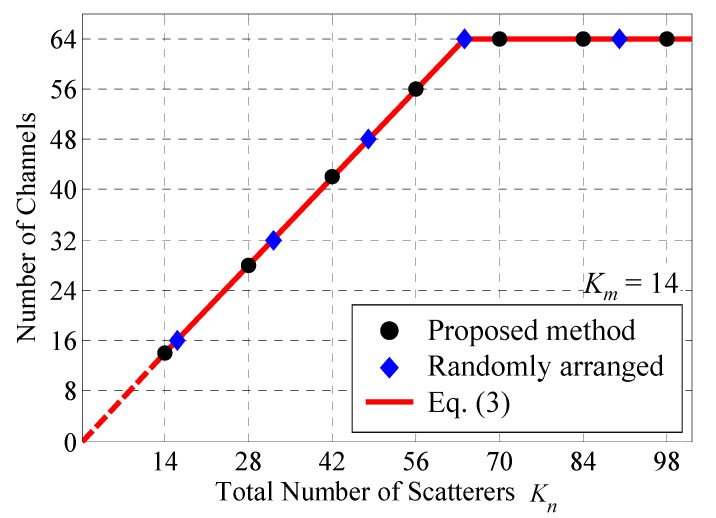
Number of channels vs. number of total scatterers.

Figure 3 shows the number of channels as a function of the total number of scatterers *K_n_*, that is the number of scatterers located to perform all measurements. In Figure 3, the black circles represent the analytical outcome with the proposed method as a function of *S*, whereas the blue rhombuses are those of the randomly arranged scatterers method, in which the scatterers on each BS were randomly selected from among all the scatterers. The red line represents the theoretical value, as expressed in Equation (3). *K_m_* is set to 14.

It can be seen from Figure 3 that the proposed method has the same effect as the method of randomly arranged scatterers. When *K_n_* is greater than 64, the number of channels equals 64. In contrast, when *K_n_* is less than 64, the number of channels is understood to be just *K_n_*. Hence, full-rank status can be achieved by rotating the actual scatterers multiple times, even with a small number of scatterers on the OTA apparatus.

**Figure 4:**
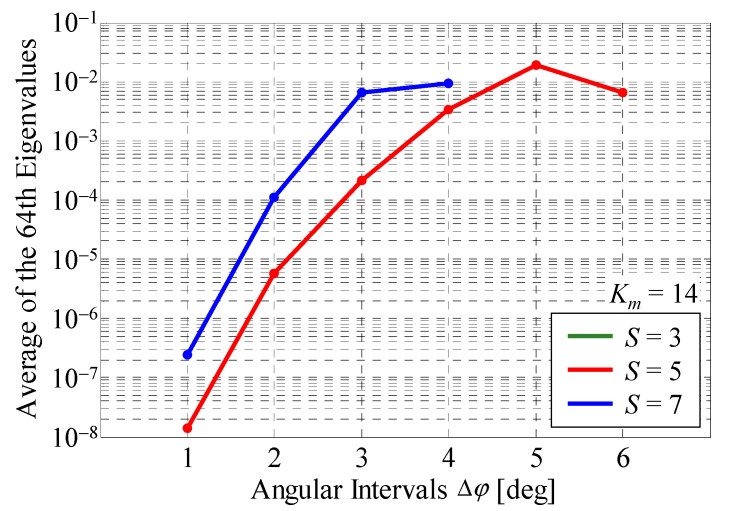
Average of the 64th eigenvalues.

Figure 4 shows the average of the 64th eigenvalues through all snapshots as a function of the angular interval between the *i*-th and (*i* + 1)-th sets of scatterers with *S* as a parameter [17]. The green, red, and blue curves indicate the cases where *S* is 3, 5, and 7, respectively. *K_m_* is set to 14.

As can be seen in Figure 4, there is no green curve for *S* = 3 because the total number of scatterers is only 42 (=14 × 3), indicating a rank-deficient status. In contrast, when *S* is greater than 5, the average of the 64th eigenvalues is confirmed, indicating full-rank status.

The average of the 64th eigenvalues is increased with increasing the angular interval. This is because, when Δ*φ* is small, there is high correlation coefficient between the incoming waves due to the closely spaced arrangement of the scatterers. On the other hand, the independence of the incoming wave is greater as Δ*φ* is increased i.e., the eigenvalues are larger. But the average of the 64th eigenvalues is reduced for more large angular intervals such as Δ*φ* = 6° with *S* = 5. In this case, the azimuth angle of scatterer #1 in the 5th set is 24°, which is close to that of scatterer #2 in the 1st set, 25.7°. Thus, all the scatterers including the virtual scatterers should be set to equal angular intervals.

### 3.2. Experimental Results

This subsection is devoted to verification of the proposed method utilizing the fading emulator with a small number of scatterers. The channel response matrix of the 64 × 64 MIMO system was measured using a two-dimensional fading emulator with a uniform incident wave distribution in azimuth. In millimeter wave 5G communications, the distance between the BS and MS is smaller than previous communication systems because the path loss between the BS and MS is large, resulting in an environment in which line-of-sight propagation or a cluster power distribution is assumed. In the sub-6 GHz frequency bands, the propagation environment is like the cluster or uniform power distributions found in previous communication systems. In this paper, a uniform power distribution, with which a sufficient number of paths in a propagation environment are expected, is considered.

**Figure 5:**
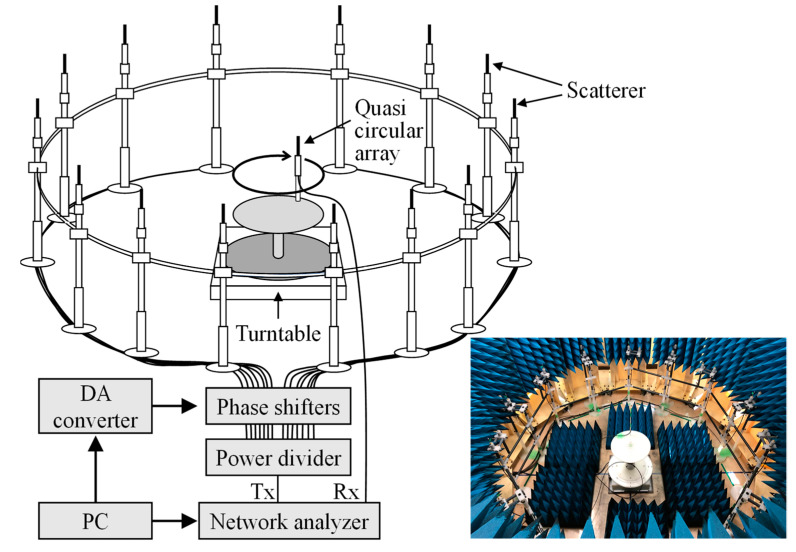
Configuration of the massive MIMO-OTA apparatus.

Figure 5 shows the configuration of the massive MIMO-OTA apparatus that embodies the proposed method using the turntable, as shown in Figure 2b. The inserted lower right photo is a bird’s eye view of the fading emulator. A 64-element quasi half-wavelength dipole MIMO circular array antenna was placed on a turntable located at the center of the fading emulator. The radius of the massive MIMO antenna was 20 cm. Fourteen scatterers, comprising vertically polarized half-wavelength sleeve dipole antennas, were set at equal angular intervals on a circle of radius 120 cm. The frequency was set to 5 GHz.

**Figure 6:**
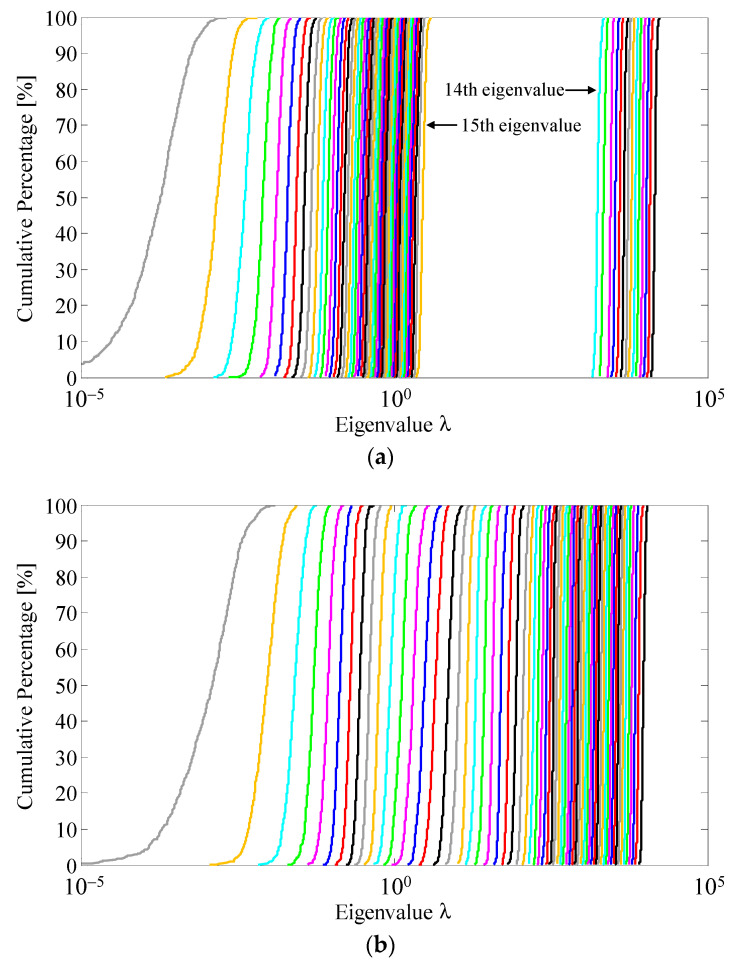
CDF characteristics of the eigenvalues: (**a**) previous method; (**b**) proposed method.

Figure 6 shows the cumulative distribution function (CDF) of the instantaneous eigenvalues obtained through SVD for a measured channel response matrix utilizing a fading emulator with a small number of scatterers. Figure 6a,b show the results for the previous method without rotation of the massive MIMO antenna and for the proposed method with rotation of the massive MIMO antenna, respectively. In the proposed method, *S* is set to 5 and the incremental rotation angle, Δ*φ*, as illustrated in Figure 1, is 5.1°, that is, the angular intervals between the scatterers are equal.

As shown in Figure 6a, there is a large interval between the 14th and 15th eigenvalues. Therefore, the previous OTA testing method can emulate only 14 channels with the same *K_m_*. However, the 15th and subsequent eigenvalues are observed. This is due to the fact that the measured channel matrix includes noise and that the time correlation is 0.999, not absolutely 1. In contrast, in Figure 6b, an extremely dense eigenvalue distribution is observed because five scatterer sets have the same effect as 70 scatterers. Consequently, a full-rank channel matrix for a massive MIMO system can be implemented using the proposed method.

**Figure 7:**
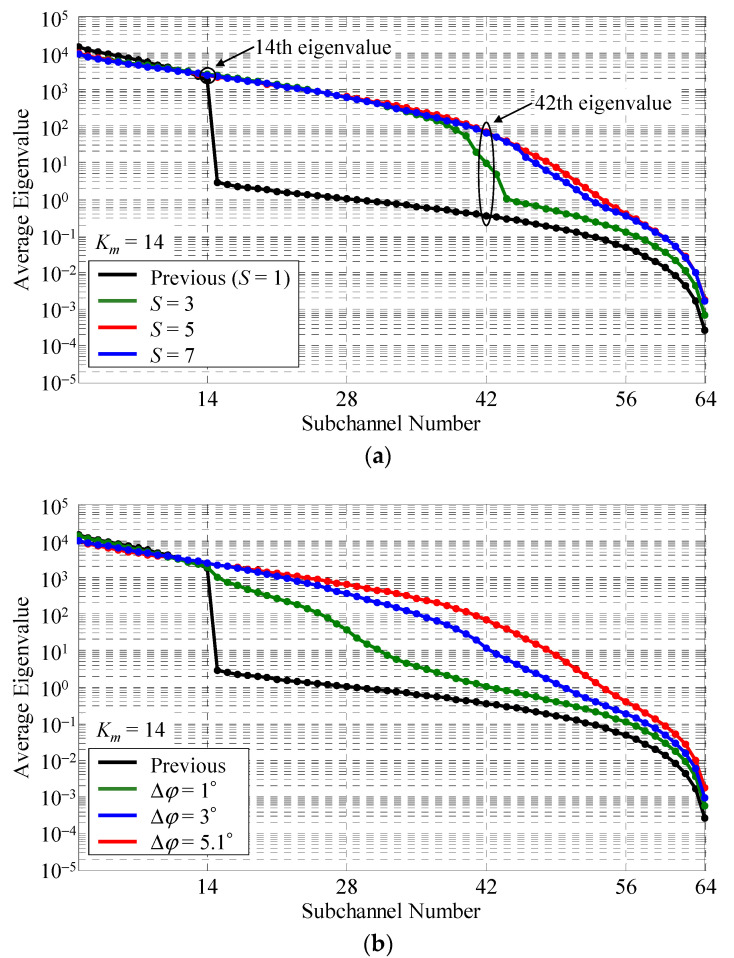
Eigenvalue distribution: (**a**) with the number of scatterer sets as a parameter; (**b**) with the rotation angle increment as a parameter.

Figure 7a shows the average eigenvalue distribution with *S* as a parameter. The green, red, and blue curves denote the cases where *S* is 3, 5, and 7, respectively. For comparison, the previous method is depicted by the black curve in the graph. When the actual scatterers were rotated, Δ*φ* was set to equal angular intervals between the *i*-th and (*i* + 1)-th sets. As the angular interval between the scatterers is 25.7° with *K_m_* set to 14, Δ*φ* is 5.1° with *S* equal to 5.

As can be seen from Figure 7a, when *S* is smaller than 3, the number of channels is less than 64 because *K_n_* is less than *M*. Specifically, the number of available eigenvalues at *S* = 1 and 3 is 14 (=14 × 1) and 42 (=14 × 3), respectively. These results agree well with those in Figure 3. In contrast, when *S* is greater than 5, the number of channels is 64. Therefore, the proposed method can achieve a full-rank channel matrix. Note that, the required number of scatterer sets is determined by the number of elements at BS, *M*, and the number of actual scatterers, *K_m_*.

Figure 7b shows the average eigenvalue distribution with Δ*φ* as a parameter. The green, blue, and red curves indicate the cases where Δ*φ* is 1°, 3°, and 5.1°, respectively. *S* is set to 5.

It can be observed that the gap in the distribution of average eigenvalues is eliminated and the distribution becomes more uniform with increasing Δ*φ*. This is because, when Δ*φ* is small, the channels are insufficiently independent owing to the proximity between the *i*-th and (*i* + 1)-th sets, resulting in the characteristics of the measured results in Figure 4. Eventually, the setting of *S* and Δ*φ* depending on *K_m_* is one of the most important issues for generating a radio propagation environment for a massive MIMO system with a large number of channels.

**Figure 8:**
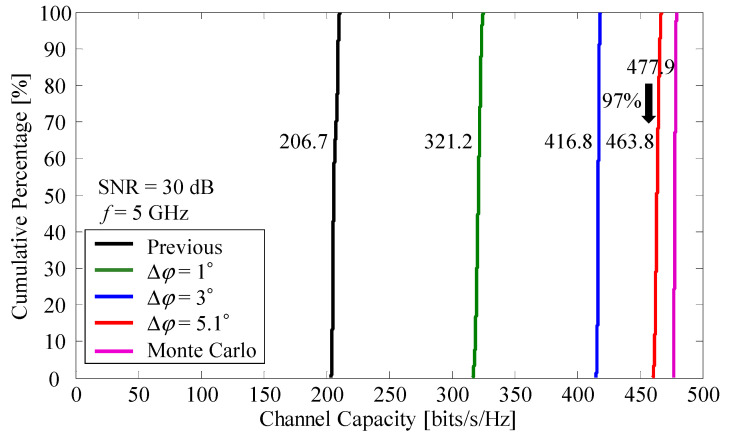
CDF characteristics of a system with 64 × 64 MIMO channel capacity.

Figure 8 shows the CDF of the instantaneous channel capacity of the 64 × 64 MIMO array as a function of Δ*φ*. The green, blue, and red curves describe the cases where Δ*φ* is 1°, 3°, and 5.1°, respectively. *S* is set to 5. For comparison, the analytical outcome through Monte Carlo simulation for the realization of a full-rank channel matrix is illustrated by the purple curve in the graph. The values shown in the graph for each case are the average channel capacity calculated from the following equation:

(5) $$\bar{C}=\frac{1}{S}\sum_{s=1}^{S}C_{s}$$

where *C_s_* indicates the channel capacity of the *s*-th snapshot and *S* is the number of snapshots. The input signal-to-noise ratio (SNR), defined as the SNR for each incident wave when a theoretical isotropic antenna is used for receiving the incident wave, is set to 30 dB. Therefore, the SNR is determined only by the received power of the isotropic antenna and is not depended on the output power of the actual BS or the network analyzer used in the fading emulator. However, since an isotropic antenna does not exist, the received power measured using other antennas must be compensated. In this paper, the power received by an isotropic antenna *REF* is calculated as follows [15]:

(6) $$REF=\frac{E\left[{\left|S_{21}\right|}^{2}\right]}{G_{d}}$$

where $E\left[{\left|S_{21}\right|}^{2}\right]$ indicates the average received power of the half-wavelength dipole antenna, placed at the center of the fading emulator, measured using the network analyzer. *G_d_* is the maximum radiation gain of half-wavelength dipole antenna in the horizontal plane, i.e., 2.15 dBi. Therefore, the SNR is determined by

(7) $$SNR=\frac{REF}{N}$$

where *N* is the power of the noise.

As shown in Figure 8, the average channel capacity increases with increasing Δ*φ*. These results can be understood from Figure 7b, because the channel capacity is calculated using the eigenvalues of the channel matrix [7]. Moreover, the channel capacity measured at Δ*φ* = 5.1° achieves 97% of the analytical outcome; indicating that the observed result corresponds to the simulation value. The channel capacity of about 450 bits/s/Hz at an SNR of 30 dB, which is equivalent to 45 Gbps with a bandwidth of 100 MHz, is fully satisfied, which is one of the most important performance goals of 5G mobile communication [1]. It is concluded from these results that OTA testing incorporating the proposed method is a valid approach for obtaining a full-rank channel matrix for a massive MIMO system.

## 4. Conclusions

This paper presents an OTA evaluation method in which a full-rank channel matrix is created for a massive MIMO MS antenna utilizing a fading emulator with a small number of scatterers. The massive MIMO MS antenna is placed at the center of a turntable which is rotated; the total number of scatterers can be determined by controlling the rotation of the massive MIMO antenna. The experimental results reveal that a full-rank channel matrix for a massive MIMO antenna system can be obtained embodying the proposed method. This is a valuable tool for assessing the high MIMO channel capacity of a massive MIMO antenna.

Future work may include verification of the proposed method for the cluster propagation environment assumed in 5G mobile communication systems.

## Data Availability

Not applicable.
